# Angiopoietin-2 Is Associated with Aortic Stiffness in Diabetes Patients in Ghana: A Case-Control Study

**DOI:** 10.1155/2023/3155982

**Published:** 2023-10-12

**Authors:** Jennifer A. Agyekum, Kwame Yeboah

**Affiliations:** ^1^Department of Physiology, University of Ghana Medical School, Accra, Ghana; ^2^Medical Laboratory Unit, Mamprobi Hospital, Ghana Health Service, Accra, Ghana

## Abstract

**Objective:**

Impaired angiogenesis, measured as serum levels of angiogenic growth factors, may be among the mechanisms underlining aortic stiffness in diabetes patients. We studied the association between aortic stiffness and circulating angiogenic growth factors in type 2 diabetes (T2DM) patients without any organ damage.

**Methods:**

In a case-control design, aortic pulse wave velocity (PWV), augmentation index (AIx), and aortic blood pressures (BPs) were measured in 140 T2DM patients and 110 nondiabetic controls. Fasting blood samples were collected to measure the levels of angiopoietin- (Ang-) 1, Ang-2, and vascular endothelial growth factor-A (VEGF).

**Results:**

Compared to nondiabetes participants, T2DM patients had increased PWV (8.7 ± 1.5 vs. 7.6 ± 1.3, *p* = 0.031), aortic pulse BP (58 ± 20 vs. 49 ± 17, *p* = 0.011), Ang-2 (838 (473–1241) vs. 597 (274–1005), *p* = 0.018), and VEGF (72.2 (28–201.8) vs. 48.4 (17.4–110.1), *p* = 0.025) but reduced levels of AIx (21.7 ± 13.8 vs. 34 ± 12.9, *p* < 0.001) and Ang-1 (33.1 (24.7–42.1) vs. 41.1 (30–57.3), *p* = 0.01). In all study participants, compared to those in the lower tertile, participants in the upper tertile of Ang-2 had increased odds of PWV (2.01 (1.17–3.84), *p* = 0.004), aortic systolic BP (1.24 (1.04–1.97), *p* = 0.011), and aortic pulse BP (1.19 (1.04–1.82), *p* = 0.041) but reduced odds of AIx (0.84 (0.71–0.96), *p* = 0.014) in multivariable-adjusted models.

**Conclusion:**

In our study population, increased circulating Ang-2 was associated with increased levels of aortic stiffness parameters.

## 1. Introduction

The prevalence of diabetes in the sub-Saharan African population is on the increase, and this is associated with early, aggressive forms of cardiovascular diseases (CVDs) [1]. Our previous studies reported increased aortic stiffness in diabetes patients in Ghana [2, 3], and aortic stiffness has been associated with the future occurrence of CVD events and mortality in diabetes patients [4]. Aortic stiffness can be measured noninvasively as the aortic pulse wave velocity (PWV) and its surrogate aortic systolic and pulse pressures. The mechanism underlining aortic stiffening in diabetes patients, leading to the deterioration of organ function, seems to be multifactorial and incompletely understood [1, 5]. Unlike peripheral blood pressures (BP) like brachial BP, aortic systolic BP is the actual pressure generated by the heart, and it is a true reflection of ventricular afterload. Aortic systolic BP is a function of cardiac factors such as stroke volume and ejection time, as well as arterial properties like stiffness and wave reflection [4]. Increased stiffness in central elastic arteries like the aorta might be a result of the degradation of elastic fibres in the lamina, causing an elevation in aortic impedance and pulse wave velocity (PWV) [5]. In addition, an increase in peripheral resistance, due to microvascular aberrations, may cause increased wave reflection, leading to elevation of the aortic augmentation index (AIx), defined as the proportion of aortic pulse pressure that is accounted for by the reflected pulse wave [6, 7]. The association between microvascular function and aortic stiffness may be a bidirectional relationship; increased aortic stiffness hampers the capacity of central elastic arteries to dampen the pulsatile BP, transmitting the pulsatile energy of BP to deteriorate the microvessels [5]. Microvascular dysfunction, likewise, increases peripheral resistance and mean BP, leading to arterial wall hypertrophy and stiffness [5]. Therefore, the predictive ability of aortic stiffness, particularly PWV, is often linked with microvascular dysfunction, such as abnormal glomerular filtration in chronic kidney disease [8] and cerebral hypoperfusion in stroke patients [9].

The integrity of microvascular function partly depends on angiogenesis, which is regulated by angiogenic factors, notably vascular endothelial growth factor (VEGF), angiopoietin- (Ang-) 1, and Ang-2 [10]. There is evidence that levels of angiogenic factors and possibly functional angiogenesis in health and disease conditions, as well as aortic stiffness, have racial variations [6, 7, 11]. Few studies have reported on the relationship between aortic stiffness and angiogenic growth factors, and none in the sub-Saharan African population. We, therefore, investigated the association between aortic stiffness and circulating angiogenic factors, Ang-1, Ang-2, and VEGF, in T2DM patients in Ghana. We hypothesize that patients with an imbalance in angiogenic growth factors are associated with increased aortic stiffness.

## 2. Materials and Methods

### 2.1. Study Design and Participants

We conducted a case-control study at the Korle-Bu Teaching Hospital in Accra, from December 2019 to June 2020. Systematic random sampling was used to select every 3^rd^ consenting patient visiting the diabetes clinic, while the nondiabetic individuals were conveniently invited from the surrounding communities to join the study. All the nondiabetic controls were screened with an oral glucose tolerance test before joining the study. Individuals with diagnosed vascular pathologies, such as nontraumatic limb amputation, vascular surgery, and known CVD patients, were excluded from the study. After applying all the eligibility criteria, a total of 250 participants, comprising 140 diabetes patients and 110 nondiabetic individuals, were included in the final analysis. Ethical approval of the study was granted by the University of Ghana Medical School Ethical and Protocol Review Committee (Protocol ID number: MS-Et/M.2–P.4.10/20122013), and all participants gave written informed consent.

### 2.2. Anthropometry and BP Measurement

A stadiometer was used to measure weight and height, and body mass index (BMI) was calculated as the ratio of weight (kg) and height squared (m^2^). We also used a measuring tape to measure waist and hip circumferences. Blood pressure was measured oscillometrically with a BP monitor (Omron 991X, Omron Health Care, Japan). Participants with a BP ≥ 140/90 mmHg and/or the use of antihypertensive medication were categorized as having hypertension.

### 2.3. Aortic Stiffness Assessment

Aortic stiffness indices such as aortic PWV, aortic systolic BP, aortic pulse pressure, and AIx were measured with the Arteriograph (TensioMed Kft., Hungary) after the participant had rested in a supine position for 10 minutes. PWV was computed as the ratio between aortic path length and the time interval between the peaks of the direct (first) and reflected (late) systolic wave (return time) [12, 13]. The aortic path length was measured with specialized callipers as the jugulum–symphysis distance. The aortic PWV was calculated as the ratio of the jugulum-symphysis distance and half of the return time.

### 2.4. Biochemical and ELISA Analysis

Fasting blood samples were collected from the antecubital vein from all study participants in the morning between 7 and 9 am to measure plasma glucose and lipid profile using a BC 300 semiautomated chemical autoanalyzer (Contec, China) and commercial reagents (Medsource Biomedicals, India). The samples were stored at -80°C until the ELISA analyses were performed. We assayed the levels of Ang-1, Ang-2, and VEGF using the commercial duoset ELISA kits (R&D Systems, Minneapolis, MN) and following the manufacturer's protocols. The assays were performed in triplicates, and the total interassay coefficient of variation for the three assays was <8%. The lowest limit of the detection is 0.18 ng/ml for VEGF, 0.16 ng/ml for Ang-1, and 0.06 ng/ml for Ang-2.

### 2.5. Statistical Analysis

**Figure 1:**
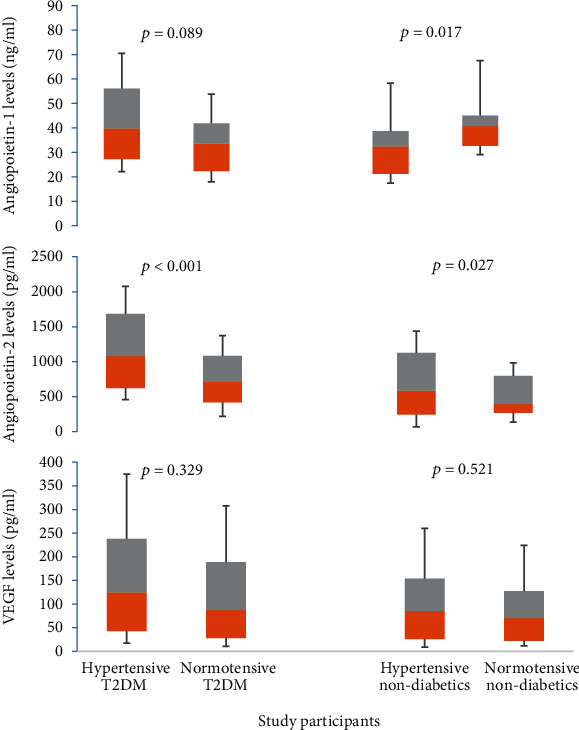
Circulating levels of angiogenic growth factors based on the hypertensive status of study participants.

Data were analysed using Jamovi 2.3.13 statistical software. The association between angiogenic growth factors and aortic stiffness indices was examined using tertile analysis. First, we examined the distribution of the various angiogenic growth factors and found that the distributions of Ang-2 and VEGF were skewed; hence, we applied a logarithmic transformation (Figure 1). Afterwards, we classified all the study participants into tertiles and compared the upper and lower tertiles. Comparison of means was performed using independent sample *t*-tests for two groups and one-way ANOVA for more than two groups. The distribution of categorical data was compared with Pearson's *χ*^2^. For the independent samples, the Kruskal–Wallis test was conducted for nonnormally distributed data. Binary and multivariable logistic regression analyses were performed to compare the odds of the change in aortic indices (PWV, AIx, aortic systolic, and pulse BPs) between the lower and upper tertiles of angiogenic growth factors (Ang-1, Ang-2, and VEGF). Also, multiple linear regression models were performed to assess the association between angiogenic growth factors and aortic stiffness indices. A *p* < 0.05 was considered statistically significant.

## 3. Results

Compared to nondiabetes controls, diabetes patients were mostly hypertensives and had higher heart rate, plasma glucose, and total and LDL cholesterol levels but lower HDL cholesterol levels. Concerning aortic stiffness indices, compared to nondiabetes participants, diabetes patients had higher levels of PWV, aortic systolic and pulse BPs, and a lower aortic augmentation index. With regards to angiogenic growth factor levels, compared to nondiabetes participants, Ang-2 and VEGF were higher in diabetes patients, but Ang-1 levels were lower (Table 1).

**Figure 2:**
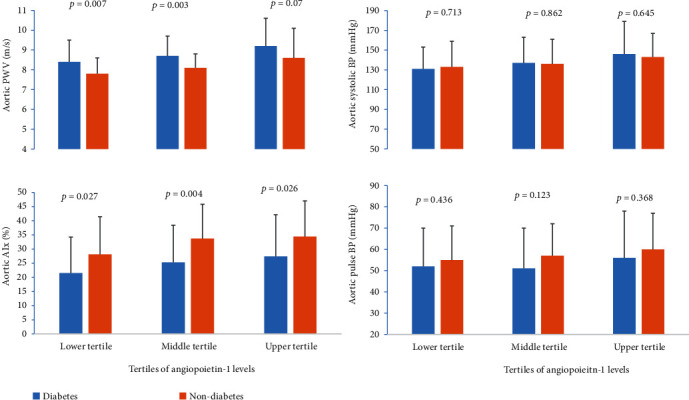
Comparison of indices of aortic stiffness between T2DM patients and nondiabetic controls among tertiles of angiopoietin-1 levels.

**Figure 3:**
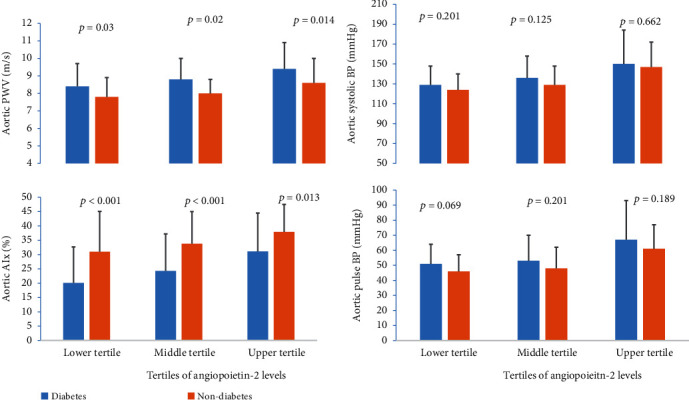
Comparison of indices of aortic stiffness between T2DM patients and nondiabetic controls among tertiles of angiopoietin-2 levels.

When study participants were categorized based on their hypertensive status, Ang-1 levels were lower in hypertensive nondiabetics compared to normotensive nondiabetics. Ang-2 levels were higher in hypertensive T2DM patients and hypertensive nondiabetics compared to their respective nonhypertensive counterparts. There was no difference in the levels of VEGF among hypertensive and nonhypertensive participants (Figure 1). When the study participants were grouped into tertiles of Ang-1 levels, compared to their nondiabetic counterparts, T2DM patients had higher aortic PWV in the lower and middle tertiles but not in the upper tertile. In all tertiles of Ang-1, T2DM patients had lower AIx levels compared to their nondiabetic controls. There was no difference in aortic systolic BP and pulse BP among various tertiles of Ang-1 (Figure 2). In tertiles of Ang-2, compared to nondiabetic controls, T2DM patients had higher aortic PWV and lower AIx in all tertiles. There was no difference in the levels of aortic systolic and pulse BPs among T2DM and nondiabetic controls (Figure 3).

The study participants were categorized based on the tertiles of angiogenic growth factor levels. In tertiles of Ang-1, compared to those in the lower tertile, PWV, aortic systolic BP, and aortic pulse BP were higher in participants in the upper tertile. In tertiles of Ang-2, compared to those in the lower tertile, PWV and aortic systolic BP were higher in participants in the middle and upper tertiles. Aortic stiffness indices across various tertiles of VEGF were not significantly different (Table 2).

In logistic regression models with participants in the lower tertile as the reference group, participants in the upper tertile of Ang-1 had increased odds of PWV and aortic systolic BP in the unadjusted model but not in the multivariable-adjusted model. Among tertiles of Ang-2, participants in the upper tertile had increased odds of PWV, AIx, aortic systolic BP, and aortic pulse BP in both unadjusted and multivariable-adjusted models. No significant change in the odds of aortic indices was observed across tertiles of VEGF (Table 3).

Multivariate regression models were constructed with the dependent variables being aortic indices (PWV, AIx, and aortic systolic BP) and the independent variables being angiogenic growth factors. From the analyses, Ang-2 was associated with PWV, aortic systolic BP, and AIx in both the age- and gender-adjusted and multivariable-adjusted models. Ang-1 was positively related to only aortic systolic BP in the age- and sex-adjusted model but not in the multivariate model. VEGF was not related to any of the indices of aortic stiffness in both models (Table 4).

## 4. Discussion

The main findings of this study were (1) diabetes patients have higher levels of arterial stiffness and Ang-2 but lower levels of Ang-1, compared to nondiabetic controls; and (2) having higher levels of Ang-2 was associated with an increase in all indices of aortic stiffness. Our previous studies have shown that the coexistence of diabetes and hypertension was associated with increased aortic stiffness [2]. Also, impaired angiogenic factors have been associated with renal dysfunction [14] and peripheral arterial disease [15] in diabetes patients. The findings of the current study may suggest that diabetes may influence aortic stiffness by causing an imbalance in angiogenic growth factors, particularly Ang-2.

The findings of this study are consistent with the levels of angiogenic factors reported in Caucasian and Asian populations. For example, in British diabetes patients, endothelial damage was associated with an imbalance in the levels of angiogenic growth factors, particularly Ang-2 and VEGF levels; no association with Ang-1 was observed [16]. In Japanese patients with hyperlipidaemia, those with diabetes had an elevation of Ang-2 levels but not that of Ang-1 [17]. In the Indian population, impaired glucose metabolism and high blood pressure were associated with Ang-2 as well [18]. Likewise, in the ASCOT studies, patients with hypertension had increased levels of Ang-1, Ang-2, and VEGF compared to the normotensives [19]. Contrary to VEGF levels reported in most studies, VEGF levels were similar among diabetes and nondiabetes participants in our study, possibly due to the ethnic variation in angiogenic growth factors, which have been reported in a comparative study involving Caucasians and African Caribbean origins [11].

Hyperglycemia in diabetes may induce an imbalance in the expressions of angiopoietins, and this may lead to the development of diabetes-related microangiopathy [20]. Due to the contrasting microvascular effects of Ang-1 and Ang-2 upon Tie-2 receptor stimulation, an imbalance of Ang-1 and Ang-2 in T2DM patients will result in disequilibrium in angiogenesis [10, 16, 21–23], leading to exuberant yet dysfunctional neovascularization in the diabetic retinopathy, as well as vascular destabilization as observed in skeletal and cardiac muscle [24]. In addition, elevated Ang-2 levels are reported to predict renal failure in chronic kidney disease patients [25, 26]; heart failure in acute myocardial infarction patients [27, 28]; and cerebral perfusion and vascular damage in murine diabetic models [29]. In diabetic nephropathy animal models, increased Ang-2 levels [30] and decreased Ang-1 [31] were associated with glomerulonephritis and glomerulosclerosis, respectively.

In this study, Ang-2 was the major angiogenic factor associated with PWV and AIx in multiple regression models. Also, participants in the upper tertiles of Ang-2 had increased odds of PWV and AIx in multivariable-adjusted models. PWV and AIx are noninvasive markers of aortic stiffness and wave reflection, respectively [6, 7]. Increased Ang-2 expression has been demonstrated to cause microvascular rarefaction in obese diabetic murine models [32, 33], explaining the utility of serum Ang-2 levels in predicting the occurrence of acute myocardial syndrome in prospective studies [27]. Microvascular rarefaction, caused by increased Ang-2 levels and presented as reduced capillary density or increased nonperfused capillaries, may lead to increased peripheral vascular resistance, causing increased wave reflection [34]. Increased wave reflection may lead to higher central systolic pressure augmentation, indicating aortic stiffness [35], as observed in this study. The association between Ang-2 and aortic stiffness in the multiple regression analysis in the study was different from what was reported in a community study with a large sample size, conducted in the Caucasian population in the United States [36]; in that study, Ang-2 was found to be negatively associated with the inverse of carotid-femoral PWV and reflection coefficient. The difference might be attributed to the different modes of aortic stiffness assessment. We used the arteriograph to measure aortic PWV in our study as opposed to the use of carotid-femoral PWV in white participants from the United States. Also, both aortic stiffness and Ang-2 have intrinsic genetic and ethnic variability [6, 7, 11, 37]. Ang-2 is implicated in vascular inflammation as reported in animal models to sensitize endothelial cells to inflammatory markers and mediate endothelial expression of adhesion molecules in vascular fibrosis in mice [38]. Also, experimental nephrectomized mice synthesize high levels of Ang-2 which stimulated the expression of proinflammatory cytokines and adhesion molecules in aortic endothelial cells, leading to enhanced collagen formation and deposition into the aortic wall [39].

## 5. Limitations and Conclusion

The major limitations of this study were the cross-sectional design which cannot infer causation, the usage of hospital patients already under treatment, and the measurement of comparatively fewer humoral factors of angiogenesis. All the same, the findings of this study have shown that in diabetes patients in Ghana with no established CVDs, an imbalance in angiogenic growth factors, particularly elevated Ang-2, may partly contribute to aortic stiffness.

## Figures and Tables

**Table 1:** General characteristics of study participants.

	All participants (*n* = 250)	T2DM (*n* = 140)	Nondiabetic controls (*n* = 110)	*p*
Females, *n* (%)	125 (50)	63 (45)	62 (56.4)	0.15
Age (yrs)	54.1 ± 10.2	53.7 ± 10.1	54.6 ± 10.3	0.54
Hypertensive	101 (40.4)	84 (60)	17 (15.5)	<0.001
Insulin medication		46 (32.9)		
Weight (kg)	79.5 ± 14.9	79.9 ± 15.5	79 ± 14.3	0.672
Height (cm)	166 ± 8.4	167 ± 8	164 ± 9	0.061
BMI (kg/m^2^)	29.1 ± 5.7	28.9 ± 5.9	29.4 ± 5.5	0.571
Waist circumference (cm)	98 ± 14	99 ± 12	96 ± 15	0.073
Waist-hip ratio	0.91 ± 0.11	0.92 ± 0.07	0.9 ± 0.14	0.382
Systolic BP (mmHg)	139 ± 30	141 ± 26	135 ± 34	0.174
Diastolic BP (mmHg)	83 ± 13	83 ± 13	82 ± 14	0.594
Pulse BP (mmHg)	59 ± 14	59 ± 14	58 ± 13	0.485
Heart rate (bpm)	71 ± 17	75 ± 13	65 ± 19	<0.01
FPG (mmol/l)	6.9 ± 3.2	8.4 ± 2.9	5 ± 2.5	<0.01
2 h-PPG (mmol/l)	7.8 ± 1.4		7.8 ± 1.4	
Triglycerides (mmol/l)	1.1 ± 0.5	1.1 ± 0.5	1.2 ± 0.6	0.586
Total cholesterol (mmol/l)	4.7 ± 1.5	5.5 ± 1.4	3.9 ± 1.1	<0.001
HDL cholesterol (mmol/l)	0.9 ± 0.2	0.7 ± 0.2	1.2 ± 0.4	0.025
LDL cholesterol (mmol/l)	3.2 ± 1.4	3.9 ± 1.3	2.7 ± 1.4	<0.001
PWV (m/s)	8.4 ± 1.4	8.7 ± 1.5	7.6 ± 1.3	0.031
AIx (%)	25.7 ± 14.2	21.7 ± 13.8	34 ± 12.9	<0.001
Aortic systolic BP (mmHg)	136 ± 26	138 ± 26	131 ± 27	0.064
Aortic PP (mmHg)	54 ± 19	58 ± 20	49 ± 17	0.011
Angiopoietin-1 (ng/ml)	40 ± 17.7	42.8 ± 21	36.4 ± 11.3	0.009
Angiopoietin-2 (pg/ml)	856 (519–1201)	893 (595–1316)	773 (483–1088)	0.018
VEGF (pg/ml)	79 (36–179)	105 (44–222)	66 (34–126)	0.036

BMI: body mass index; BP: blood pressure; FPG: fasting plasma glucose; 2 h-PPG: 2-hour postglucose load plasma glucose; HDL: high-density lipoprotein, LDL: low-density lipoprotein; PWV: aortic pulse wave velocity; AIx: aortic augmentation index; VEGF: vascular endothelial growth factor.

**Table 2:** Comparison of aortic stiffness indices across various tertiles of angiogenic growth factors.

	Lower tertile	Middle tertile	Upper tertile	*p*
*Angiopoietin-1*				
PWV	8.3 ± 1.1	8.4 ± 1.6	8.9 ± 1.5^∗^	0.019
AIx	26.4 ± 14.1	28.3 ± 15.1	29.8 ± 14.3	0.096
Aortic systolic BP	132 ± 24	135 ± 21	144 ± 29^∗^	0.013
Aortic PP	50 ± 17	55 ± 16	58 ± 20^∗^	0.025
*Angiopoietin-2*				
PWV	8.1 ± 1.3	8.7 ± 1.2^∗^	9.3 ± 1.4^∗^	0.017
AIx	24.1 ± 14.4	28.5 ± 15.2	33.9 ± 12.4^∗^	<0.001
Aortic systolic BP	131 ± 23	138 ± 22	149 ± 29^∗^	0.001
Aortic PP	49 ± 17	53 ± 14	60 ± 21^∗^	0.005
*VEGF*				
PWV	8.4 ± 1.5	8.7 ± 2.1	8.6 ± 1.4	0.313
AIx	26.7 ± 14	27.9 ± 15.6	30.6 ± 14.3	0.144
Aortic systolic BP	133 ± 22	135 ± 29	139 ± 26	0.202
Aortic PP	52 ± 15	55 ± 13	56 ± 18	0.27

BP: blood pressure; PWV: aortic pulse wave velocity; AIx: aortic augmentation index; VEGF: vascular endothelial growth factor. ^∗^*p* < 0.05 compared to the lower tertile.

**Table 3:** Logistic regression models comparing aortic stiffness indices of participants in the upper tertile of angiogenic growth factors to those in the lower tertile.

	Crude OR (95% CI)	*p*	Adjusted OR (95% CI)^∗^	*p*
*Angiopoietin-1*				
PWV	1.42 (1.07–1.69)	0.007	1.27 (0.96–1.87)	0.066
AIx	1.03 (0.99–1.1)	0.211	1.01 (0.97–1.05)	0.73
Aortic systolic BP	1.19 (1.04–1.35)	0.001	1.02 (0.97–1.07)	0.488
Aortic PP	1.08 (1.01–1.19)	0.033	1.03 (0.98–1.08)	0.255
*Angiopoietin-2*				
PWV	2.83 (1.23–4.01)	<0.001	2.01 (1.17–3.84)	0.004
AIx	1.53 (1.13–1.84)	0.001	0.84 (0.71–0.96)	0.014
Aortic systolic BP	1.24 (1.09–1.4)	0.002	1.24 (1.04–1.97)	0.011
Aortic PP	1.31 (1.09–1.53)	0.006	1.19 (1.04–1.82)	0.041
*VEGF*				
PWV	1.06 (0.82–1.37)	0.636	0.93 (0.67–1.28)	0.929
AIx	1.02 (0.99–1.05)	0.141	1.03 (0.98–1.08)	0.405
Aortic systolic BP	1.01 (0.99–1.03)	0.238	1.03 (0.99–1.08)	0.81
Aortic PP	1.01 (0.99–1.03)	0.339	1.01 (0.96–1.09)	0.812

BP: blood pressure; PP: pulse pressure; PWV: aortic pulse wave velocity; AIx: aortic augmentation index; VEGF: vascular endothelial growth factor. ^∗^adjusted for age, gender, diabetes, and hypertension status, BMI, and mean BP.

**Table 4:** Association between angiogenic growth factors and indices of aortic stiffness from multiple linear regression models.

Aortic indices	Growth factor	Age- and sex-adjusted model	*p*	Multivariable model^∗^	
		*β*		*β*	*p*
PWV	Ang-1	0.069	0.32	-0.016	0.82
	Ang-2	0.162	0.03	0.148	0.03
	VEGF	0.006	0.93	-0.01	0.89
AIx	Ang-1	0.039	0.58	0.032	0.63
	Ang-2	0.147	0.03	0.188	<0.01
	VEGF	0.033	0.96	0.073	0.28
Systolic BP	Ang-1	0.156	0.03	0.049	0.51
	Ang-2	0.274	<0.01	0.222	<0.01
	VEGF	0.057	0.44	0.07	0.33

All of the angiogenic growth factors are logarithmically transformed. Ang-1: angiopoietin 1; Ang-2: angiopoietin 2; VEGF: vascular endothelial growth factor; BP: blood pressure; PWV: aortic pulse wave velocity; AIx: aortic augmentation index. ^∗^Multivariable models were adjusted for age, sex, diabetes status, hypertension status, body mass index, heart rate, total cholesterol, triglycerides, and mean blood pressure. *β*: standardized regression coefficient.

## Data Availability

The data set supporting the conclusion of this study is available for systematic review and meta-analysis upon request.

## Abbreviations

PW: Aortic pulse wave velocity
AIx: Aortic augmentation index
BP: Blood pressure
Ang: Angiopoietin
VEGF: Vascular endothelial growth factor.
